# Electrochemical insights into the mechanism of NiFe membrane-bound hydrogenases

**DOI:** 10.1042/BST20150201

**Published:** 2016-02-09

**Authors:** Lindsey A. Flanagan, Alison Parkin

**Affiliations:** * Department of Chemistry, University of York, Heslington, York YO10 5DD, U.K.; † 2013 Early Career Research Award, Theme Panel III Energy and Metabolism

**Keywords:** iron–sulfur cluster relay, membrane-bound hydrogenase, NiFe hydrogenase, oxygen tolerance, protein film electrochemistry

## Abstract

Hydrogenases are enzymes of great biotechnological relevance because they catalyse the interconversion of H_2_, water (protons) and electricity using non-precious metal catalytic active sites. Electrochemical studies into the reactivity of NiFe membrane-bound hydrogenases (MBH) have provided a particularly detailed insight into the reactivity and mechanism of this group of enzymes. Significantly, the control centre for enabling O_2_ tolerance has been revealed as the electron-transfer relay of FeS clusters, rather than the NiFe bimetallic active site. The present review paper will discuss how electrochemistry results have complemented those obtained from structural and spectroscopic studies, to present a complete picture of our current understanding of NiFe MBH.

## Introduction

**Figure 1:**
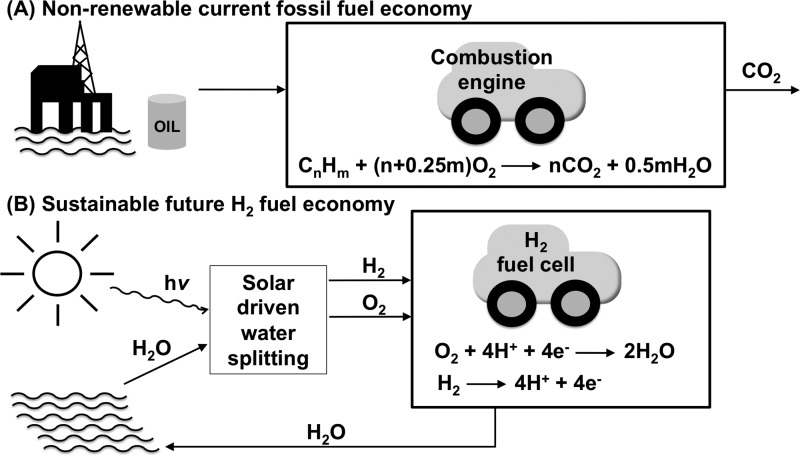
Cartoon depicting the contrast between (A) the non-renewable, current fossil fuel economy, and (B) a sustainable, future H_2_ fuel economy.

Humankind's current fossil fuel economy is unsustainable: it is non-renewable, generates the greenhouse gas carbon dioxide and relies on finite resources which are not evenly distributed across the globe, creating geo- and political- access issues [1]. As depicted in Figure 1, in comparison with the use of fossil fuels, a renewable H_2_ fuel economy presents many advantages: generating H_2_ from water is a cyclical, sustainable process, and vehicular H_2_ technology is a commercial reality [2]. The challenge lies in finding redox catalysts for the half cell processes of proton reduction (2H^+^+2e^−^ → H_2_) and water oxidation (H_2_O → ½O_2_+2H^+^+2e^−^) to achieve the overall reaction of solar driven water splitting (H_2_O+*hν* → H_2_+½O_2_) [3]. There is a requirement for highly efficient catalysts built from commonly available elements that combine protons and electrons to produce H_2_.

Hydrogenases are H_2_ enzymes that are produced by a wide variety of microbes to catalyse either H_2_ splitting or the reverse reaction, H_2_ production [4,5]. As biological catalysts, hydrogenases are stable in water, built from earth-abundant elements and have high substrate affinities and fast turnover rates [6], and these combined factors have fuelled an interest into how hydrogenases can be utilized within a future H_2_ economy. Understanding how hydrogenases function as molecular H_2_ catalysts is of fundamental importance to any biological-H_2_ technology development and this paper will highlight how electrochemistry, in conjunction with other techniques, has played a vital role in deconvoluting the mechanism of NiFe hydrogenases.

## Hydrogenases

There are three main types of hydrogenases, named for the composition of their active site as the NiFe, FeFe and Fe only hydrogenases [7]. The majority of biotechnological hydrogenase enzyme-devices, whether fuel cells or H_2_ producing devices, have made use of one subclass of NiFe hydrogenases, the Group 1 membrane-bound hydrogenases (MBH) [8,9]. These enzymes are biotechnologically useful because they react reversibly with O_2_, whereas the FeFe and Fe only hydrogenases sustain permanent damage after reaction with O_2_. Many MBH also adsorb onto carbon surfaces in an electrocatalytic configuration, generating a heterogeneous catalyst of “wired” enzyme molecules without the need for complex surface modification.

**Figure 2:**
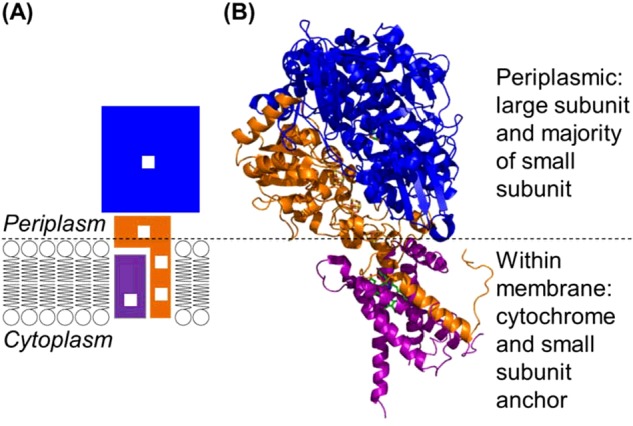
The orientation of a NiFe MBH within a bacterial cell (**A**) Cartoon depiction of how a NiFe MBH is located within the cytoplasmic membrane, with white boxes representing the redox active metal centres and blue, orange and purple blocks indicating the large, small and cytochrome subunits, respectively. (**B**) Crystallographic insight into how the *E. coli* hydrogenase-1 large (blue ribbon), small (orange ribbon) and cytochrome (purple ribbon) subunits can interact. Figure generated from PDB 4GD3 [68].

The NiFe MBH will be the focus of this paper, although reference will be made to soluble periplasmic enzymes in order to clarify aspects of the reactivity. Membrane-bound hydrogenases are periplasmically located enzymes which are embedded in the inner membrane of a bacterial cell [10,11], as shown in Figure 2. The physiological function of NiFe MBH is H_2_ uptake, the conversion of H_2_ into protons and electrons (H_2_ → 2H^+^+2e^−^). The electrons are transferred from the hydrogenase into the quinone pool, with the NiFe MBH therefore forming part of the bacterial respiratory chain [12]. NiFe MBH are found in bacteria from a diverse range of ecological niches, from human pathogens such as *Salmonella enterica*, in which hydrogenase activity is linked to virulence [13], to soil bacteria like *Ralstonia eutropha*, which can survive by using H_2_ as their sole energy source [14], and *Allochromatium vinosum*, a photosynthetic purple sulfur bacteria which uses light energy to oxidize hydrogen sulfide to elemental sulfur [15].

The NiFe MBH are sub-categorized into enzymes which are able to function in O_2_ and enzymes which do not, with the former classed as O_2_ tolerant while the latter are known as O_2_ sensitive MBH [16]. The O_2_ reactivity of hydrogenases is considered important because most water splitting technology requires an O_2_ insensitive H_2_-catalyst [17]. Additionally, O_2_ tolerant NiFe MBH have been used to develop membrane-free H_2_ fuel cells, powered by non-explosive H_2_/O_2_ mixes [9]. Such devices are amenable for miniaturization because of their simple design.

In addition to highlighting the utility of electrochemistry as a technique for studying NiFe MBH, the present paper also will review our current mechanistic understanding of what controls the reaction of a hydrogenase with O_2_.

## Hydrogenase film electrochemistry

### Protein film electrochemistry applied to hydrogenases

Protein film electrochemistry, a technique in which enzyme is adsorbed to the surface of an electrode, has been a particularly useful tool for interrogating the reactivity of NiFe MBH. The Armstrong Group at the University of Oxford has played a major role in pioneering this field of research [18,19]. In these studies, the hydrogenase molecules under interrogation are commonly the “minimal functional unit” of an MBH, i.e. the active site (large) and electron-transfer (small) subunits, since these often purify separately from other subunits [20]. The possible impact of this is returned to later in the present paper (Section ‘The cytochrome: beyond the dimeric unit of a NiFe membrane-bound hydrogenase’). Although the enzyme molecules are assumed to be randomly orientated on the electrode surface, the electroactive orientations–where redox cofactors are in close enough proximity to the electrode to facilitate rapid electron transfer [21]–are thought analogous to the “wiring” of the hydrogenase to the cytoplasmic membrane.

**Figure 3:**
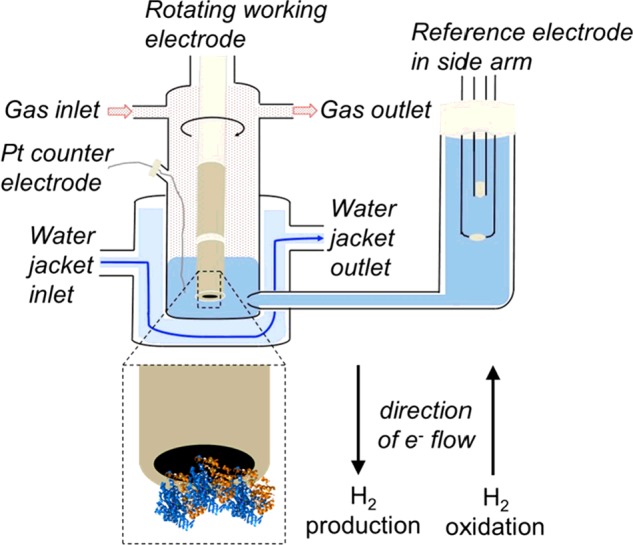
Diagram of the electrochemical cell with detail of how the direction of electron flow in/out of the working electrode changes depending whether H_2_ production (H^+^ reduction, an electron uptake reaction) or H_2_ oxidation (an electron producing reaction) is catalysed by the hydrogenase adsorbed on the electrode surface.

The electrochemical cell, shown in Figure 3, is designed to facilitate temperature control via the water jacket, and pH control is determined by the buffer composition of the experimental solution within the main body of the cell. There are three separate electrodes, the working electrode, onto which the hydrogenase is adsorbed; the reference electrode, which is the reference point against which the voltage of the working electrode is set; and the counter/auxiliary electrode, which completes the electrical circuit by “countering” the electron flow at the working electrode. The working electrode is capable of rotation, which allows the investigation and minimization of diffusion effects, such as product inhibition. Gas flow controllers upstream of the electrochemical cell allow precise control of headspace gas composition, and mixtures of H_2_, N_2_, and the inhibitors O_2_ and CO can be made.

**Figure 4:**
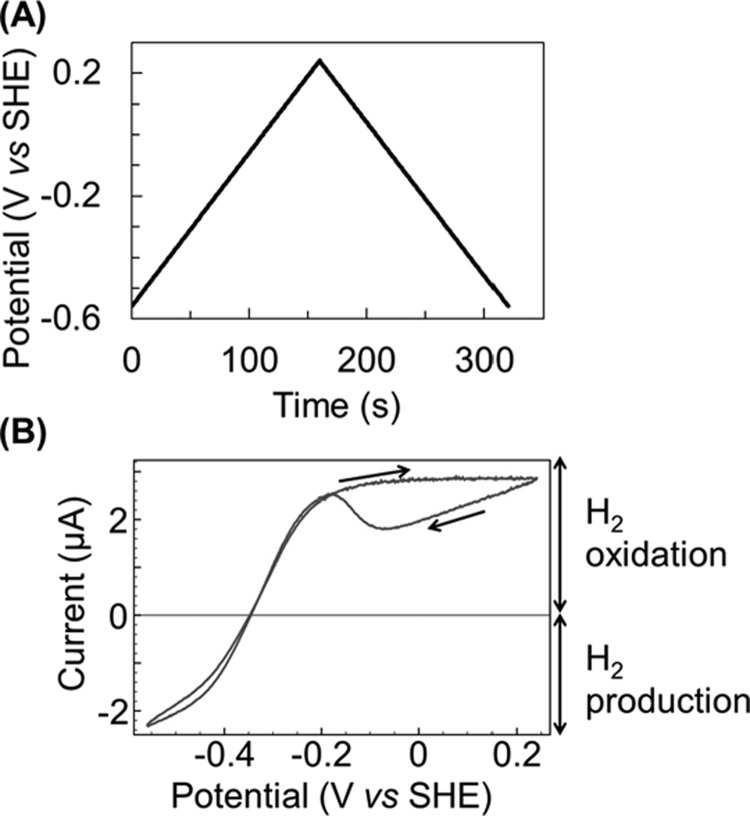
How cyclic voltammetry can be used to measure the bidirectional H_2_ catalytic activity of a hydrogenase under a H_2_-containing gas atmosphere (**A**) The potential–time linear sweep applied to the hydrogenase-coated working electrode, and (**B**) the resultant current–potential response. The enzyme used in this example is *E. coli* Hyd-2.

The bidirectional catalytic activity of NiFe hydrogenases is clearly revealed in cyclic voltammetry experiments such as that shown in Figure 4. The electrochemical potential of the hydrogenase-coated electrode is raised from negative (reducing) potentials to positive (oxidizing) potentials and the resultant current is monitored. Electrical current corresponds to enzymatic activity, with negative current providing a direct measure of H_2_ production (when electrons are transferred from the electrode into the enzyme as in Figure 3) and with positive current measuring H_2_ oxidation (where the direction of electron flow is reversed, also shown in Figure 3). If the number of enzyme molecules adsorbed onto the surface of the electrode is known (the product of the electrode surface area, *A*, and the surface density of enzyme on the electrode, Γ) then the catalytic current, *i_cat_*, can be converted into a turnover rate, *k_cat_*, via eqn (1), where *n* denotes the number of electrons, 2 in the case of H_2_ catalysis, and *F* is Faraday's constant [18].

1 $$\;i_{cat}=k_{cat}\times nFA\Gamma$$

In most studies it has not been possible to measure Γ as the number of enzyme molecules on the electrode surface is often too low to produce the necessary non-catalytic signals needed to determine accurate coverage [22]. Therefore, electrochemistry is not commonly used to measure hydrogenase turnover rates, instead dye assays are used to quantify hydrogenase rate constants [23]. For enzyme-catalysed H_2_ oxidation, the concomitant reduction in either methylene blue or benzyl viologen can be followed spectrophotometrically [24]. Alternatively, the rate of H_2_ production/H^+^ reduction can be followed by measuring the rate of oxidation of reduced methyl viologen. A significant limitation of the dye assays is that they do not work in the presence of O_2_ and electrochemistry has provided an unparalleled comparison of the reactions of hydrogenases under anaerobic compared with aerobic conditions.

### Electrochemical definition of hydrogenase O_2_ tolerance

**Figure 5:**
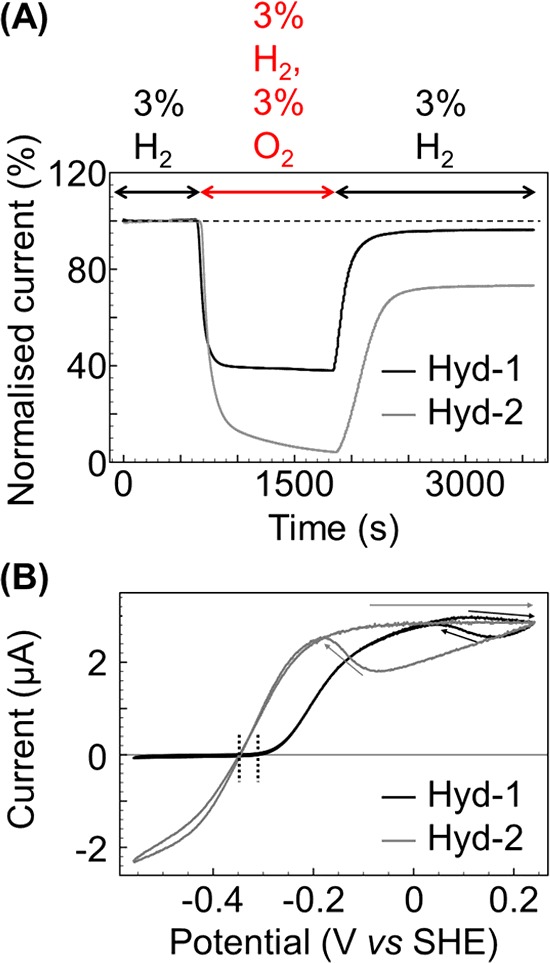
Contrasting the electrochemical signature of an O_2_ tolerant NiFe MBH (black line, *E. coli* Hyd-1) with that of an O_2_ sensitive isozyme (grey line, *E. coli* Hyd-2) under (A) O_2_ inhibition conditions and (B) O_2_-free conditions (**A**) Constant potential experiments measuring the percentage of initial H_2_ oxidation activity which is sustained when the enzyme is exposed to O_2_, and also quantifying the reversibility of the inhibition. For Hyd-1 a voltage of +0.06 V versus SHE is used, for Hyd-2 -0.16 V versus SHE. (**B**) 5 mV s^−1^ cyclic voltammetry experiments highlighting the difference in catalytic activity of O_2_ tolerant and sensitive NiFe MBH under conditions of 3% H_2_. The dotted vertical bars mark the potential onset of H_2_ oxidation and the arrows indicate regions of Ni-B formation and reactivation. Other experimental conditions: pH 6, 37°C, rotation rate 3000 rpm and total gas flow rate of 100 scc min^−1^ with N_2_ as carrier gas.

Electrochemistry is particularly effective at categorizing a hydrogenase as either O_2_ sensitive or O_2_ tolerant [25]. Figure 5(A) shows a chronoamperometry experiment where the potential of the electrode is poised at a constant value and the current is measured as a function of time. The phenotypic behaviour of an O_2_ tolerant hydrogenase is that upon exposure to a mixture of H_2_ and O_2_, a substantial amount of the H_2_ oxidation activity (positive current) observed in the absence of O_2_ is maintained [26]. Upon removal of the O_2_, the electrical current returns to almost 100% of the initial activity, indicating full re-activation of the enzyme. This is shown by the “Hyd-1” data in Figure 5(A), measured for *Escherichia coli* hydrogenase-1 (Hyd-1). Conversely, O_2_ sensitive enzymes such as *E. coli* hydrogenase-2, “Hyd-2” in Figure 5(A), are fully inhibited (current tends towards zero) under O_2_-containing gas mixtures [18,26]. When O_2_ is removed from the experimental setup, not all of the enzyme activity is recovered within a short timeframe (the experiment shown in Figure 5A allows a reactivation period of 30 min).

As well as the characteristic differences in O_2_ reactivity, electrochemistry also reveals notable heterogeneity between O_2_ tolerant and O_2_ sensitive hydrogenases in the absence of O_2_, as shown by Figure 5(B). Firstly, measurement of the catalytic activity via cyclic voltammetry shows that whereas enzymes which are tolerant to O_2_ produce very little H_2_ at pH levels above 6 (there is negligible negative current for Hyd-1 in Figure 5B), the ratio of maximum negative current to maximum positive current is almost 1:1 for O_2_ sensitive hydrogenases at near-neutral pH [26]. We can summarize this observation by stating that the catalytic “bias” is different for O_2_ tolerant and sensitive MBH; the O_2_ tolerant enzymes are essentially unidirectional H_2_-uptake enzymes at pH >6 while the O_2_ sensitive hydrogenases are bidirectional H_2_ catalysts over a wide pH range (5–8) [10]. The crucial role of pH in controlling the H_2_ production activity of O_2_ tolerant hydrogenases will be described later on.

A second observation related to catalysis is that O_2_ sensitive hydrogenases are “ideal” H_2_-catalyts; the potential of zero current (the voltage at which there is no net H_2_ oxidation or H_2_ production activity) equates to the reduction potential for the 2H^+^/H_2_ couple under the experimental conditions, *E*(2H^+^/H_2_) [26] (the value of *E*(2H^+^/H_2_) is calculated using the Nernst equation and the standard reduction potential value *E*^Θ^(2H^+^/H_2_)=0 V versus SHE). In contrast with this ideal behaviour, O_2_ tolerant hydrogenases such as Hyd-1 manifest an “overpotential requirement” at pH >5, meaning that the potential at which H_2_ oxidation catalysis commences is more positive than for Hyd-2 [27]. This is highlighted by the vertical dotted lines in Figure 5(B), which show that the onset of H_2_ oxidation is ∼50 mV higher for Hyd-1 than for Hyd-2.

It is helpful to correlate the differences in reactivity measured by electrochemistry with structural insights gained by spectroscopic and crystallographic studies, in order to build a mechanistic understanding of the difference between O_2_ tolerant and O_2_ sensitive MBH. The next sections will describe how all these data can be combined to build a picture of hydrogenase structure–function properties.

## Active site states

### States formed in the absence of O_2_

**Figure 6:**
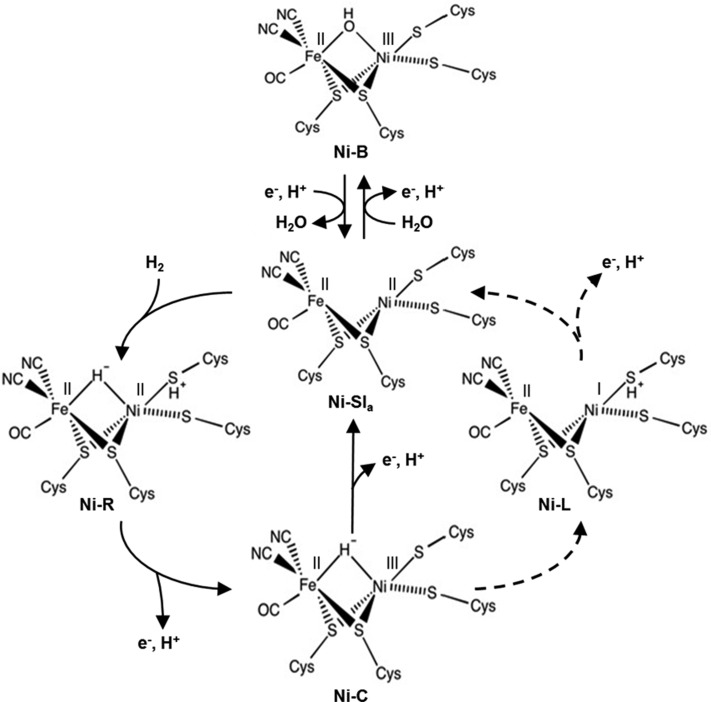
Catalytic and inhibited active site redox states accessed by NiFe hydrogenases under O_2_-free conditions The solid arrows indicate the reaction mechanism for O_2_ sensitive NiFe hydrogenases. The dashed arrows indicate how the Ni-L state may participate as a reaction intermediate in the catalytic cycle of O_2_ tolerant MBH.

Detailed structural information about the active site of NiFe MBH has been obtained from spectroscopic measurements using a variety of techniques including EPR [28] and IR [29–32]. A summary of the proposed key states in the catalytic cycle is given by Figure 6; for simplicity this figure shows uni-directional H_2_ oxidation, although the same states are proposed to occur for H_2_ production/H^+^ reduction.

When catalysing H_2_ oxidation, NiFe hydrogenases operate via a heterolytic cleavage mechanism whereby the H_2_ initially binds to the Ni-Si_a_ (Ni^II^–Fe^II^) state as a proton and a hydride to form the most reduced Ni-R (Ni^II^–H^−^–Fe^II^) state of the active site (Figure 6) [33]. Loss of a proton and an electron then generates the Ni-C (Ni^III^–H^−^–Fe^II^) state. At this stage in the H_2_ uptake reaction the proposed mechanisms for O_2_ tolerant and O_2_ sensitive enzymes differ, with EPR and FTIR studies showing that different active site states predominate within each hydrogenase subgroup. For O_2_ sensitive NiFe hydrogenases the Ni-L (Ni^I^–Fe^II^) state is not thought to be a true catalytic intermediate because it is only formed under light exposure at very low temperatures [34–36]. As denoted by the solid arrows in Figure 6, catalysis by O_2_ sensitive NiFe hydrogenases is thought to proceed via loss of an electron and proton from the Ni-C state to give direct regeneration of Ni-SI_a_. In contrast, for O_2_ tolerant hydrogenases the Ni-L state is readily detectable at room temperature, even when the enzyme has not been illuminated [31], so this state is considered an important catalytic intermediate. Figure 6 shows a putative Ni-C to Ni-L conversion that involves electron and proton rearrangement; Ni^III^ is reduced to Ni^I^ with concomitant movement of the bridging hydrogen to the sulfur of a coordinating cysteine.

Under conditions of oxidative stress, but in the absence of O_2_, all NiFe MBH form the inactive Ni-B state, of which the structure is well established [29,30], as shown in Figure 6. Formation of the Ni-B state is also thought to be observable in cyclic voltammetry electrochemistry experiments, such as those shown in Figures 4 and 5(B). Under O_2_-free conditions, when the potential is steadily increased above -0.2 V for Hyd-2 or 0 V for Hyd-1, a current plateau is observed. This means that the H_2_ oxidation activity levels off despite the application of more oxidizing potentials. This is denoted by the arrows pointing from left to right in Figure 5(B). Upon reversal of the potential sweep, so that the voltage of the electrode is now being made progressively more negative, the MBH is activated, as shown by the sharp rise in current from -0.1 to -0.2 V for Hyd-2 and +0.15 to +0.05 V for Hyd-1, and highlighted by the diagonal arrows in Figure 5(B). Comparison of electrochemically measured inactivation and reactivation kinetics with spectroscopic studies has led to attribution of the current plateau and recovery to formation and reactivation of the Ni-B state, respectively [25,37]. The observation that all O_2_ tolerant hydrogenases apparently require an additional oxidative driving force to form the Ni-B state in cyclic voltammetry experiments has been used as the basis for a model for understanding how these enzymes remain catalytically active in the presence of O_2_ [38]. However, there is nothing about the active site structures which explains why formation of the Ni-B state should require a different electrochemical potential for O_2_ tolerant compared with O_2_ sensitive enzymes [29,38]. Modern research has focused on understanding how the movement of electrons within the hydrogenase controls the active site chemistry, and this is described later on.

### States formed by reaction with O_2_

The Ni-B state is also formed when NiFe MBH are exposed to O_2_. The mechanism of this conversion is still controversial. One proposed reaction [39] involves O_2_ binding at the active site followed by reduction by four electrons and three protons to form water and a hydroxide molecule (O_2_+3H^+^+4e^−^ → H_2_O+OH^−^). The water molecule formed from O_2_ is then proposed to diffuse out of the enzyme via a solvent channel, while the hydroxide remains bound to the oxidized active site. Envisaging Ni-B formation as arising from O_2_ binding at the active site has been used to reconcile the observation that O_2_ tolerant hydrogenases have decreased sensitivity to CO and very low Michaelis constants for H_2_; it has been interpreted that these enzymes have evolved to optimize H_2_ binding at the active site in order to minimize competitive inhibitor ligation at the NiFe centre [26,40].

However, the notion that aerobic Ni-B formation involves a long lived active site species formed from O_2_ has been challenged by isotope studies showing evidence that the bridging OH^−^ ligand in Ni-B is solvent derived [41], and oxygenic species originating from O_2_ are not observable in the first coordination sphere of the nickel [42]. Based on these studies, alternative mechanisms which do not involve the binding of O_2_ to the active site have been proposed. For example, O_2_ could prompt oxidation of the active site by acting as an electron acceptor, as proposed by Hamdan et al. in their work which proves that “aerobic” inactivation can occur under O_2_-free oxidizing conditions [43].

**Figure 7:**
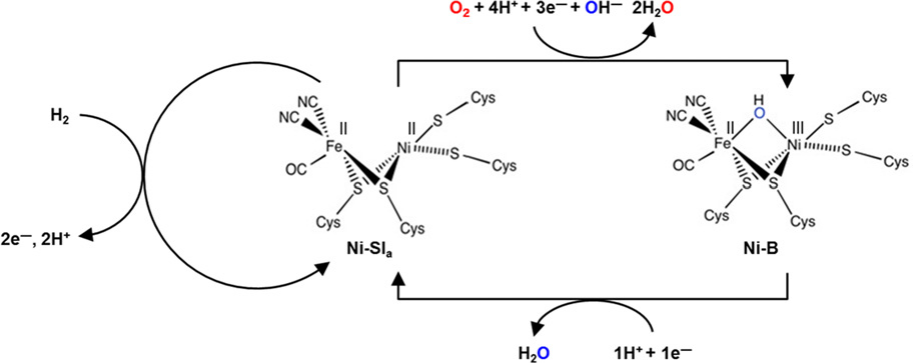
Reaction of an O_2_ tolerant NiFe MBH with O_2_ to generate the Ni-B state.

Regardless of the precise site of O_2_ binding, it is known that an O_2_ tolerant NiFe hydrogenase exposed to O_2_ can catalyse H_2_ oxidation and also act as an O_2_-reductase, converting O_2_ into two molecules of H_2_O (Figure 7) [44]. The rapid reactivation of Ni-B explains why O_2_ tolerant hydrogenases sustain H_2_ catalytic activity in the presence of O_2_; any enzyme molecule which forms the Ni-B state upon inactivation by O_2_ is quickly reactivated by addition of one electron, as shown in Figure 7. Electrochemical experiments reveal that the “*E*_switch_” potential, a complex voltammetric parameter used to characterize the reactivation window of a hydrogenase, is positive for an O_2_ tolerant MBH, reflecting the ease of Ni-B reduction [45]. For O_2_ sensitive enzymes, *E*_switch_ has a negative potential, but slower Ni-B activation is not the only reason that these enzymes are inactivated by O_2_ exposure.

**Figure 8:**
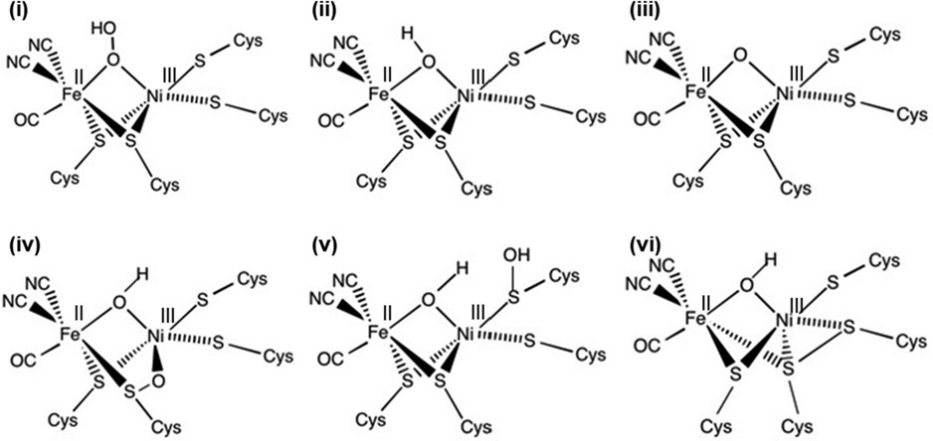
Different postulated structures for the Ni-A inhibited active site state (**i**) Bridging peroxo structure [48]; (**ii**) stereoisomer of the Ni-B, hydroxide bridged, active site [41]; (**iii**) bridging oxo structure [41]; (**iv**) Ni and S bridging oxo species [53]; (**v**) S–OH and bridging hydroxide form [54] and (**vi**) disulfide bond containing hydroxide-bridging structure [54].

In the case of O_2_ sensitive NiFe hydrogenases, spectroscopic experiments using a variety of techniques all conclude that reaction with O_2_ generates both the Ni-B state and a very kinetically inert “Unready”, Ni-A, state [46–50]. This correlates with the electrochemical observation that following O_2_ exposure, O_2_ sensitive enzymes such as Hyd-2 rapidly recover some activity as soon as the O_2_ is removed from the experiment, but a substantial proportion of the enzyme remains inactivated (Figure 5A). The rapid reactivation is attributed to recovery of molecules which formed the Ni-B state, while slow reactivation is assigned to enzyme which formed the Ni-A state [43,49]. A significant challenge in discovering the precise mechanism for Ni-A formation originates from the difficulty in reconciling the spectroscopic and electrochemical experiments with the fact that crystallographic experiments have isolated a myriad of different structures, all denoted as possible representatives of the Ni-A state (Figure 8) [48,51–54]. Due to the observation of extended electron density about the bridging ligand, early structural studies proposed that the Ni-A state contains an O_2_ derived peroxide group bridging the Ni and Fe [48] (Figure 8i). This model has been revised in recent years to consider the possibility of oxidation of the S group of cysteine ligands, as in several structures proposed by Volbeda et al. [53,54] and shown in Figures 8(iv)–8(vi). A bridging oxo group, O^2−^, was also postulated, as shown in Figure 8(iii). Carepo et al. [41] used ^17^O labelled water, H_2_^17^O, to confirm that there is a bridging ligand but it is solvent derived. The isotope labelling study instead proposed that the Ni-A state could contain a bridging hydroxide in a different orientation to that seen in Ni-B (Figure 8ii). This is supported by recent work by Barilone et al. [55] who used single crystal ENDOR spectroscopy to confirm the Ni-A bridging ligand as a hydroxide, again suggesting that the difference in the structure of Ni-B and Ni-A is due to rotation at the nickel, specifically identifying a cysteine side chain as the mobile element. Altogether, the issue of the structural identity of Ni-A remains contentious, rendering the task of explaining why O_2_ sensitive NiFe hydrogenases are slow to reactivate very difficult.

The lack of consensus in determining exactly what active site structure is generated when O_2_ sensitive NiFe hydrogenases react with O_2_ may be in part attributable to the fact that O_2_ inactivation can also generate “dead” states of permanently inactivated enzyme [19]. In electrochemistry it is difficult to separate an estimate of dead phase formation from “film loss”, the normal steady drop in enzyme activity which is observed over long experiments and is probably due to desorption of enzyme from the electrode [56]. If the hydrogenase dead phase is non-paramagnetic then it will also be rendered spectroscopically silent via EPR, and small numbers of molecules are hard to detect using FTIR. However, at least some of the structural electron density maps may reflect dead phase enzyme and so reconciling X-ray data and spectroscopic information could remain challenging.

## The essential role of the electron-transfer relay

### Description of the iron–sulfur “wire”

**Figure 9:**
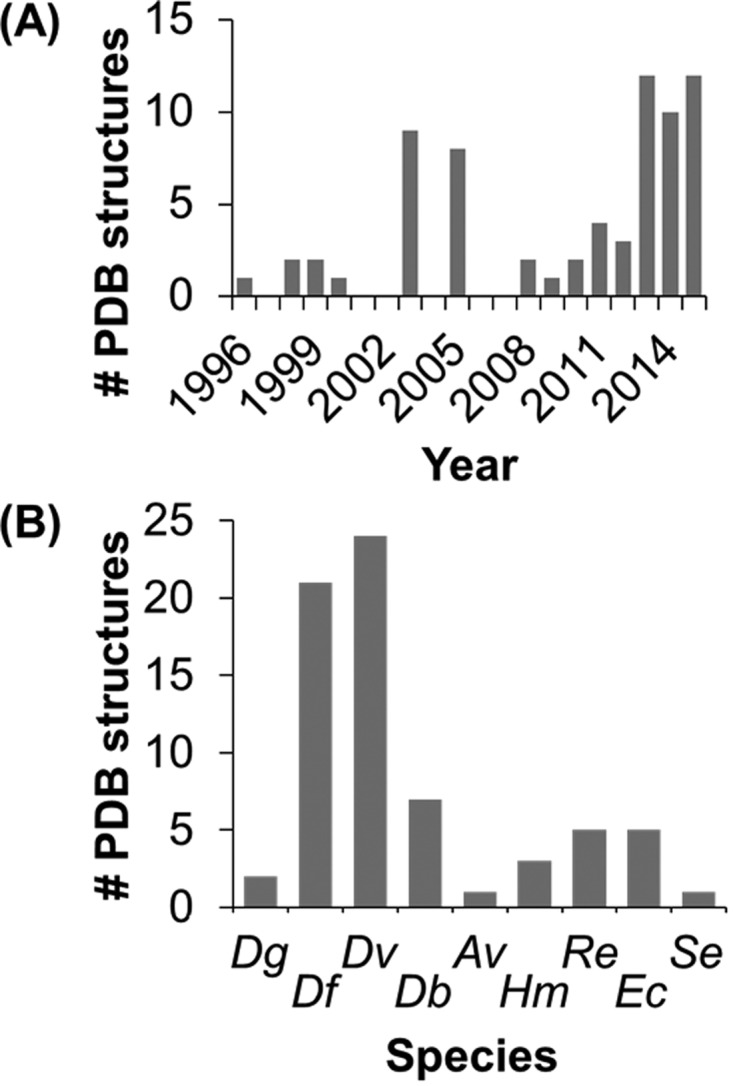
Graphs relating number of NiFe hydrogenase PDB structures to (A) publication year and (B) bacterial species Abbreviations: *Dg*, *D. gigas*; *Dv*, *D. vulgaris*; *Av*, *A. vinosum*; *Re*, *R. eutropha*; *Se*, *S. enterica*; *Df*, *D. fructosovorans*; *Db*, *D. baculatum*; *Hm*, *H. marinus*; *Ec*, *E. coli*. Generated from data in the Protein Data Bank [71].

**Figure 10:**
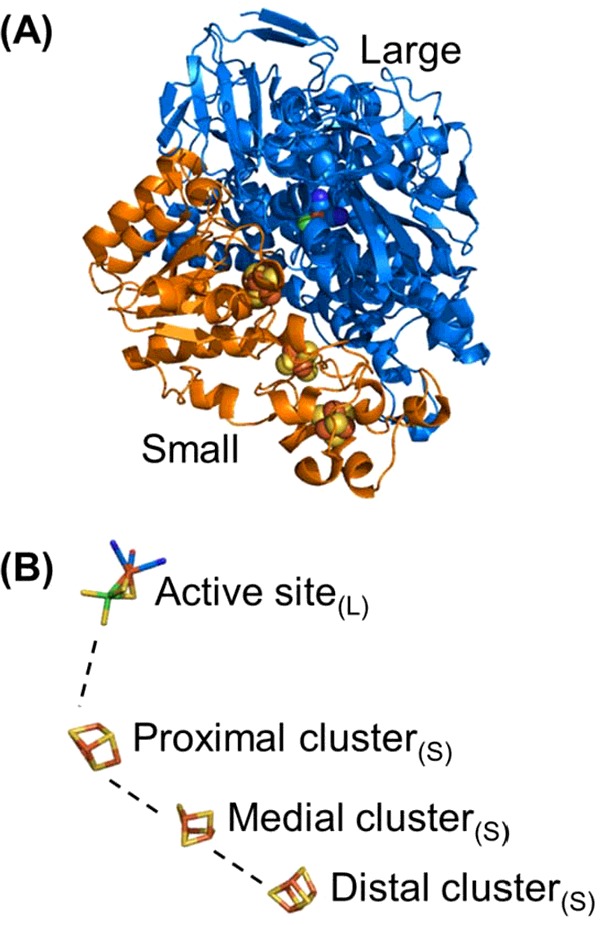
(A and B) The minimal functional unit of a NiFe hydrogenase (PDB 3RGW [60]) comprising a NiFe active site co-ordinated by the large subunit (blue ribbon) and three FeS clusters co-ordinated by the small subunit (orange ribbon).

The minimal functional unit of a NiFe MBH is a heterodimer comprising two protein chains designated the “large” and “small” subunits [57]. As shown by Figure 9(A), over the past 10 years substantial progress has been made in the determination of NiFe hydrogenase X-ray structures. Enzymes from both aerobic (*Hydrogenovibrio marinus* [58] and *R. eutropha* [59,60]), anaerobic (*Desulfovibrio gigas* [48,51], *Desulfovibrio fructosovorans* [53,54,61], *Desulfovibrio vulgaris* [52,62–64], *Desulfomicrobium baculatum* [65,66] and *A. vinosum* [67]) and facultative anaerobic organisms (*E. coli* [68,69] and *S. enterica* [70]) have been crystallized (Figure 9B) [71], resulting in the elucidation of the heterodimer structures of several O_2_ sensitive and O_2_ tolerant NiFe hydrogenases. The overall structure of all these enzymes is remarkably similar (Figure 10). In addition to the NiFe active site centre (contained within the ∼60 kDa large subunit), three iron–sulfur (FeS) clusters are ligated by the ∼35 kDa small subunit [58,59]. This “wire” of FeS clusters is thought to function as an electron-transfer relay, shuttling electrons from the buried active site to the surface of the protein, thus allowing the passage of electrons between the active site and the inner membrane [72]. The three clusters are commonly denoted as “proximal”, “distal” or “medial” in reference to their distance from the active site.

As discussed above, electrochemistry reveals clear differences in the reactivity of O_2_ tolerant and O_2_ sensitive MBH. The overpotential-requirement and uni-directional activity of the O_2_ tolerant hydrogenases may be linked to their propensity to form the Ni-L state as part of the catalytic cycle (Figure 6) but nothing about the active site architecture suggests why the Ni-L state might be more accessible. Instead, the active site binding pocket is highly conserved for both O_2_ tolerant and sensitive MBH. Recent studies have revealed that it is the electron-transfer clusters which play a critical role in tuning the reaction mechanism of the hydrogenases, and this will now be explained in detail.

### Proximal cluster

**Figure 11:**
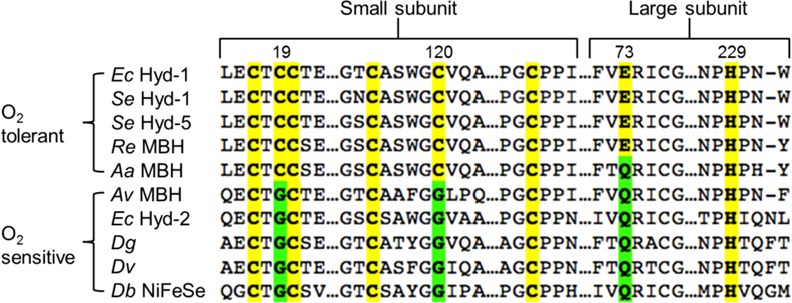
Sequence alignment highlighting how O_2_ tolerant and O_2_ sensitive NiFe hydrogenases differ in the small and large subunit amino acids located near to the proximal cluster *Ec* (*E. coli*) Hyd-1 numbering is used, this is the same as *Se* Hyd-1/Hyd-5. Other abbreviations are: *Se*, *S. enterica*; *Re*, *R. eutropha*; *Aa*, *A. aeolicus*; *Av*, *A. vinosum*; *Dg*, *D. gigas*; *Dv*, *D. vulgaris*; *Db*, *D. baculatum.*

**Figure 12:**
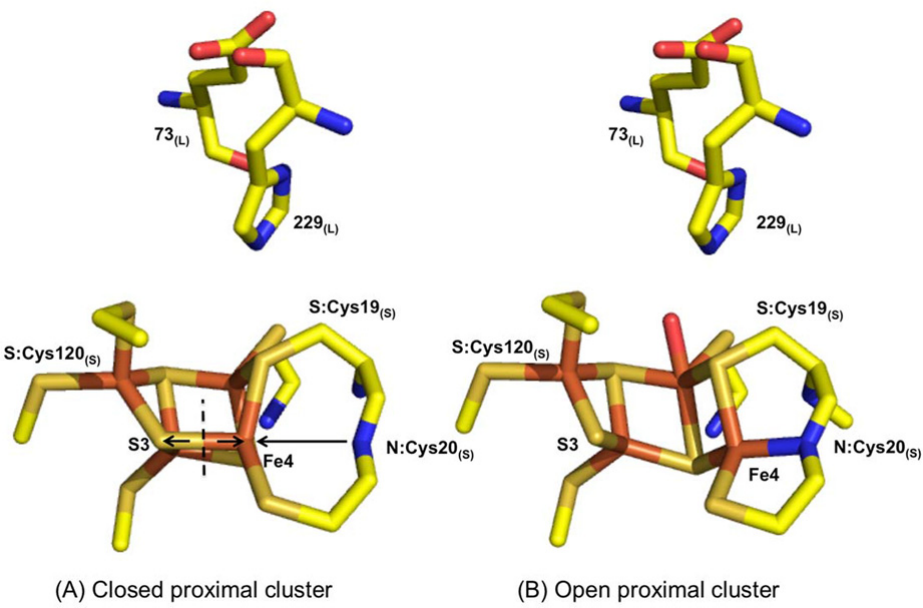
Structural insight into the oxidative “opening” of the proximal cluster obtained from *R. eutropha* NiFe MBH crystallography studies (PDB reduced=3RGW and oxidized=4IUB) [60] The cluster “opening” is ascribed to the breaking of the bond between S3 and Fe4, indicated by the dashed line and outward arrows in (**A**), and formation of a bond between Fe4 and the backbone N of cysteine 20, indicated by the arrow pointing from N:Cys20 to Fe4 in (**A**). Colours: orange=iron, ochre=sulfur, yellow=carbon, blue=nitrogen, red=oxygen. *E. coli* hydrogenase-1 / *S. enterica* Hyd-5 residue numbering is used.

Experiments on a number of different O_2_ tolerant hydrogenases have revealed that it is a uniquely structured FeS cluster that confers O_2_ tolerance upon NiFe MBH [59,72,73]. Crystallography has shown that while O_2_ sensitive NiFe hydrogenases contain a standard 4Fe4S cluster proximal to the active site, all O_2_ tolerant MBH contain a novel 4Fe3S cluster in this position [58,59]. As highlighted in the sequence alignment in Figure 11, additional cysteines are contained within O_2_ tolerant MBH, and these assist in the stabilization of the unusual proximal cluster. These “supernumerary” cysteines support a structural transition which occurs when the cluster is oxidized, for example when the enzyme is exposed to O_2_. During this transition, the backbone N of cysteine 20 ligates Fe4 of the cluster, and the bond between Fe4 and S3 of the cluster is broken, thus creating an “open” structure [60], as seen in Figure 12. This “over oxidation” can also be observed in EPR studies which show that the 4Fe3S cluster can undergo two redox transformations, i.e. it is stable in three different oxidation states: fully reduced, [4Fe3S]^3+^; oxidized, [4Fe3S]^4+^; and over oxidized, [4Fe3S]^5+^ [60,69,72,74].

Electrochemical studies on hydrogenase variants show that using glycine to replace the supernumerary cysteine which supports the cluster opening mechanism (*E. coli* Hyd-1 numbering: C19G) turns an O_2_ tolerant hydrogenase into an enzyme which is highly sensitive to O_2_ [75]. This suggests that formation of the over oxidized state of the proximal cluster plays an important role in O_2_ tolerance. Returning to Figure 7, the idea is that because the proximal cluster can access three oxidation states it is therefore able to rapidly deliver two of the electrons which are needed to ensure that inhibitory O_2_ is rapidly neutralized to H_2_O and OH^−^ [44], ensuring that the Ni-B state is the only product of reaction between an O_2_-tolerant hydrogenase and O_2_.

**Figure 13:**
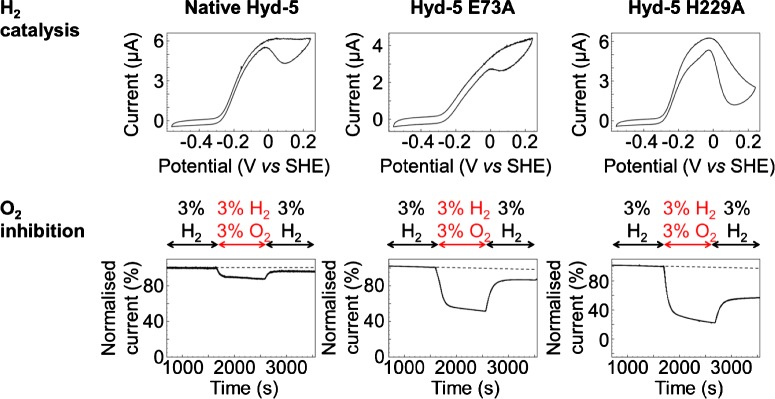
Electrochemical characterization of the catalytic (top) and O_2_ inhibition properties (bottom) of different proximal cluster variants (left to right) of *S. enterica* Hyd-5 The 5 mV s^−1^ cyclic voltammetry H_2_ catalysis experiments were measured under a gas atmosphere of 10% H_2_ while the chronoamperometry O_2_ inhibition experiments were conducted at a potential of +0.06 V versus SHE with changing gas atmosphere as indicated. Other experimental conditions: pH 6, 37°C, rotation rate 4000 rpm and total gas flow rate of 100 scc min^−1^ with N_2_ as carrier gas.

More recent work has shown that large subunit residues also play a vital role in the proximal cluster chemistry [60,70]. The sequence alignment shown in Figure 11 demonstrates that all O_2_ sensitive hydrogenases possess a glutamine in the large subunit, while the majority of O_2_ tolerant MBH encode a glutamate at this site. Structural analysis indicates that this residue is too removed from the proximal cluster to play a direct role in stabilizing the 3Fe4S redox transitions. However, in conjunction with a conserved histidine as an acid/base pair, a possible proton transfer mechanism was hypothesized and tested for variants of the O_2_ tolerant MBH *S. enterica* hydrogenase-5 (Hyd-5) [70]. While exchange of *S. enterica* Hyd-5 glutamate 73 for alanine had a minimal effect on catalysis in the absence of O_2_, in the presence of O_2_ the enzyme exhibited decreased O_2_ tolerance relative to the native enzyme (Figure 13). The essential role of E73 in tuning O_2_ tolerance was therefore confirmed. Replacing histidine 229 with alanine had a substantial effect on the H_2_ oxidizing ability of the enzyme under anaerobic and aerobic conditions. The increase in O_2_ sensitivity in large subunit histidine variants was also observed in *R. eutropha* MBH [60] and crystallographic data from that study provided evidence for a direct proton transfer route from the large subunit to the proximal cluster via H229, which points direct at one of the cluster's Fe centres, as shown in Figure 12. The role of this proton transfer pathway in triggering the structural and redox transition of the proximal cluster into the over oxidized state has been elaborated through recent density functional theory studies. Dance [76] suggests that O_2_ binding at the active site induces a change in protonation which is communicated to the proximal cluster via a proton transfer relay which ends at H229. Because the change in histidine coordination induces the proximal cluster to open up, the proton transfer pathway therefore ensures that O_2_ binding at the active site is rapidly followed by electron delivery from the proximal cluster.

Interestingly, in all proximal cluster variants to date, although it has been possible to turn an O_2_ tolerant hydrogenase into an O_2_ sensitive enzyme, the bi-directional catalytic activity of a true O_2_ sensitive NiFe MBH was not conferred upon any of the single site variants [60,70,75]. Instead, it is the distal cluster which is thought to hold the key to understanding the origins of the O_2_ tolerant over-potential requirement [77], while the medial cluster is thought to play a role in supporting the O_2_ tolerance mechanism [78].

### Medial cluster

Studies of the proximal cluster highlighted that this site was capable of providing two electrons to reduce inhibitory O_2_ [69,74], however if O_2_ attacks a Ni^II^ oxidation state of the enzyme, then generation of the Ni-B state requires a total of three electrons from the FeS relay [44] (and one from the oxidation of the active site, forming Ni^III^) (see Figure 7). Given that electrons one and two can be supplied by the proximal cluster, the most obvious source of the third FeS relay electron is the medial cluster. In both O_2_ tolerant and sensitive MBH the medial cluster is a 3Fe4S centre, ligated by three cysteines. Evans et al. [78] generated and electrochemically studied a variant of *E. coli* Hyd-1 which contained a cysteine in position 242 instead of proline. The amino acid exchange caused the medial cluster to become a 4Fe4S centre with a concomitant increase in O_2_ sensitivity. The loss of O_2_ tolerance probably arises because the medial redox potential has become more negative as a result of the amino acid exchange. This will cause the medial cluster to lose electrons to the distal cluster too readily, rather than storing them in order to reduce the proximal cluster.

A combined proximal and medial cluster variant, *E. coli* Hyd-1 C19G/C120G/P242C, showed a complete and irreversible loss of H_2_ oxidation activity following O_2_ exposure, supporting the proposed mechanism that three electrons are needed from the medial and proximal cluster in order to achieve O_2_ tolerance [78].

### Distal cluster

The distal cluster of the NiFe MBH remains the most elusive FeS centre. Although midpoint redox potentials have been quoted for *Aquifex aeolicus* [72], *R. eutropha* and *Ralstonia metallidurans* [79], it is not possible to determine the same parameter from analogous EPR experiments on *E. coli* Hyd-1. Indeed, the very careful study on Hyd-1 instead concluded that the distal cluster does not form an S=½ state even under reducing conditions [74,75]. Measuring the reduction potential of the Hyd-1 distal cluster using electrochemistry instead of EPR has so far proved impossible because of our inability to obtain “non-turnover” signals for O_2_-tolerant NiFe MBH. However, simulation of hydrogenase electrocatalytic cyclic voltammetry data supports the notion of the distal cluster controlling catalytic bidirectionality via its “gateway” role in mediating intermolecular electron transfers [77].

At low pH, substantial H_2_ production has been observed for the O_2_ tolerant hydrogenase *E. coli* Hyd-1, and there is a loss of the overpotential requirement for catalysis [27]. This result is interpreted in terms of the electrocatalysis voltammetric simulation model [77]; because the redox potential of the 2H^+^/H_2_ couple is strongly pH dependent while the distal cluster reduction potential is thought to be pH independent, there is a threshold pH below which it is thermodynamically favourable for electrons to transfer from the distal cluster into the active site thus facilitating H^+^ reduction to generate H_2_ [27,77].

The only study of a distal cluster NiFe hydrogenase variant was conducted on an O_2_ sensitive enzyme at high pH [80]. That study shows that NiFe hydrogenases are non-functional without a correctly ligated distal cluster because when the normal Cys_3_His coordination was converted to Cys_4_ or Cys_3_Gly a substantial drop (≈ 95%) in H_2_ oxidation rates was observed for *D. fructosovorans* variants. Imidazole was observed to recover the activity of the variants, emphasizing the vital role of the histidine ligand at the distal cluster.

### The cytochrome: beyond the dimeric unit of a NiFe membrane-bound hydrogenase

In a bacterial cell, a NiFe MBH is anchored on the periplasmic face of the inner membrane by a trans-membrane helix on the C-terminus of the small subunit (Figure 2) [10]. This helix is believed to associate with a hydrogenase cytochrome which embeds into the membrane [81]. The physiological electron-transfer chain of a hydrogenase does not, therefore, end at the distal cluster and it may be dangerous to interpret the in vivo role of a NiFe MBH without considering the mediating role which the cytochrome and quinone pool will play. There is, at the date of writing, only one MBH crystal structure which incorporates the cytochrome subunit (PDB 4GD3 [68]). This *E. coli* Hyd-1 structure contained a 2:1 ratio of large–small dimer:cytochrome, which is likely an experimental artefact since the operon of all NiFe MBH contain a single gene for each of the large, small and cytochrome subunits, all under control of the same promoter. Figure 2 therefore shows a fragment of the X-ray structure with one small, large and cytochrome subunit, this combination of subunits is classed as the heterotrimeric unit.

All the electrochemical experiments described above were conducted on dimers of the large and small subunits only, because the cytochrome easily dissociates during enzyme purification. However, it has been possible to isolate the O_2_ tolerant MBH from *R. eutropha* as a unit of three heterotrimers [81]. Electrochemical experiments have been conducted to analyse the behaviour of the trimeric complex embedded within a membrane-coated electrode, with a ubiquinone pool acting to shuttle electrons between the electrode and the hydrogenase [82]. In this configuration, the enzyme's electrochemical response is dramatically different from that of an isolated dimer on a membrane-free electrode. When the electrode wiring was mediated via the quinone pool, the hydrogenase trimer showed little to no anaerobic inactivation and a higher level of activity was sustained in the presence of O_2_. It has been proposed that “short-circuiting” multiple hydrogenases by forming multimers of trimers should enhance O_2_ tolerance because an active site which has H_2_ bound can provide the electrons to reduce a Ni-B inactivated neighbouring active site. There may therefore be a physiological benefit for hydrogenase molecules associating with one another within the cytoplasmic membrane. It is important to consider that while biotechnological applications of isolated dimeric enzymes require an understanding of the catalytic features of the same molecules, knowing how to harness hydrogenase activity within whole cell applications (such as cyanobacterial photosynthetic H_2_ production) may require an alternative approach such as the study of MBH within a membrane. Spectroscopic methods have also been developed to permit analysis of MBH within cell membranes [30], but there is a requirement for hydrogenase over-expression which is not always feasible because of the complex biosynthesis of these enzymes.

## Conclusion

In conclusion, when used in combination with spectroscopy and crystallography, electrochemistry has been proved an extremely useful tool for investigating NiFe MBH reactivity. Enormous progress has been made in recent years to understand the origin of O_2_ tolerance in MBH, and the essential roles of redox centres and residues remote from the NiFe active site have been elucidated and understood. There remain some core aspects of NiFe MBH biochemistry, such as the origin of catalytic bias and the structure of the Ni-A state, formed when O_2_ sensitive enzymes react with O_2_, which remain elusive. Beyond the membrane-bound Group 1 NiFe hydrogenases, there are four further categories of NiFe hydrogenases which are woefully under-studied but now becoming accessible due to modern molecular biology techniques. It is hoped that hydrogenase research will continue to contribute to the vital field of sustainable H_2_ production, inspiring the development of stable non-precious metal catalysts with high turnover frequency. It is noted that some synthetic catalysts can already outperform the enzymes under certain conditions, and purifying hydrogenases is a costly process. However, the biological synthesis of these highly active NiFe catalysts under conditions of ambient temperature, pressure and in an aqueous solvent remains an inspirational goal, and it may be that whole cell technology is where microbial catalysts are best deployed.

## Abbreviations

FTIR: Fourier transformed infrared
Hyd-1: hydrogenase-1
Hyd-2: hydrogenase-2
Hyd-5: Hydrogenase-5
MBH: membrane-bound hydrogenase(s)
